# PROSPERO at one year: an evaluation of its utility

**DOI:** 10.1186/2046-4053-2-4

**Published:** 2013-01-15

**Authors:** Alison Booth, Mike Clarke, Gordon Dooley, Davina Ghersi, David Moher, Mark Petticrew, Lesley Stewart

**Affiliations:** 1 Centre for Reviews and Dissemination, University of York, Alcuin B Block, Heslington, York, YO10 5DD, UK; 2 Centre for Public Health, Institute of Clinical Sciences, Block B, Queen’s University Belfast, Royal Victoria Hospital, Grosvenor Road, Belfast, BT12 6BA, UK; 3 Metaxis Ltd, Elmbank Offices, Elmbank Court, Main Road, Curbridge, Oxford, OX29 7NT, UK; 4 Research Translation Branch, National Health & Medical Research Council, 16 Marcus Clarke Street, Canberra City, ACT 2600, Australia; 5 Department of Epidemiology, Clinical Epidemiology Program, Ottawa Hospital Research Institute, 725 Parkdale Avenue Ottawa, Ottawa, ON, K1Y 4E9, Canada; 6 Community Medicine, Faculty of Medicine, University of Ottawa, 451 Smyth Road, Ottawa, ON, K1H 8M5, Canada; 7 Department of Social and Environmental Health Research, London School of Hygiene and Tropical Medicine, Keppel Street, London, WC1E 7HT, UK

**Keywords:** Systematic review protocol, Register, Prospero, Evaluation

## Abstract

**Background:**

PROSPERO, an international prospective register of systematic review protocols in health and social care, was launched in February 2011. After one year of operation we describe access and use, explore user experience and identify areas for future improvement.

**Methods:**

We collated administrative data and web statistics and conducted an online survey of users’ experiences.

**Results:**

On 21 February 2012, there were 1,076 registered users and 359 registration records published on PROSPERO. The database usage statistics demonstrate the international interest in PROSPERO with high access around the clock and around the world. Based on 232 responses from PROSPERO users (response rate 22%), almost all respondents found joining and navigation was easy or very easy (99%); turn round time was good or excellent (96%); and supporting materials provided were helpful or very helpful (80%). The registration fields were found by 80% to be relevant to their review; 99% rated their overall experience of registering with PROSPERO as good or excellent. Most respondents (81%) had a written protocol before completing the registration form and 19% did not. The majority, 136 (79%), indicated they completed the registration form in 60 minutes or less. Of those who expressed an opinion, 167 (87%) considered the time taken to be about right.

**Conclusions:**

The first year of PROSPERO has shown that registration of systematic review protocols is feasible and not overly burdensome for those registering their reviews. The evaluation has demonstrated that, on the whole, survey respondents are satisfied and the system allows registration of protocol details in a straightforward and acceptable way. The findings have prompted some changes to improve user experience and identified some issues for future consideration.

## Background

PROSPERO, an international database of prospectively registered systematic reviews in health and social care, was launched in February 2011. The aim of the register is to help reduce unplanned duplication of reviews, provide transparency and to help minimise reporting bias by enabling comparison of reported review findings with what was planned in the protocol [1]. PROSPERO is funded through the National Institute for Health Research in the UK and is free to register and free to search.

Researchers provide key features from their review protocol which are recorded and maintained as a permanent record in PROSPERO. The registration form contains 22 required fields and 18 optional fields, agreed through international consultation [2]. ‘Required’ fields contain ownership details and key protocol methods, such as participants, outcomes and analyses; they must be completed before a registration form can be submitted [3]. ‘Optional’ fields provide more administrative information, such as review team members and their affiliations and dissemination plans.

PROSPERO was designed to collect and process registration details accurately while keeping the process of registration as straight forward as possible in order to minimise work for researchers registering their systematic reviews.

After one year of operation, an evaluation of the registration process was undertaken to identify areas for improvement and further development. This paper outlines the evaluation and findings and discusses issues raised.

## Methods

Data relating to registered users, submitted registration forms, the administration process and web statistics for access and usage were collated for the period 22 February 2011 to 21 February 2012. Feedback and suggestions for future development were sought from those who had submitted registration requests within the same time frame of interest using electronic questionnaires, which were prepared in SurveyMonkey®, Palo Alto, CA, USA. (http://www.surveymonkey.com). The survey questions are listed in Additional file 1.

Emails containing the link to the survey were sent out to 1,076 registered users on 27 February 2012 with a reminder sent out 12 March 2012. The survey was closed on 21 March 2012. There were 29 emails returned with permanent failure to deliver messages. There were also 39 named individuals who had ‘joined’ more than once using different email addresses and who accounted for 87 registered users. As all distinct email addresses were included in the survey, some people will have received invitations to participate at more than one email address. However, SurveyMonkey was set to permit only one response per computer to minimise multiple responses from the same person. The number of individual registered users receiving the survey was 1,009.

## Results

### Registration data

#### Registration activity

On 21 February 2012, there were 359 published records on PROSPERO. Of these, 339 were on-going, 15 had been completed but not yet published and 3 had been completed and published [4-6], subsequent to registration. Two were updates of existing reviews previously registered.

In the same time period, a total of 89 submissions were ineligible for inclusion in PROSPERO and not accepted for registration (Table 1). Of these, 37 were already completed and 33 were too far advanced (progressed beyond data extraction). Nine were methodological reviews with no direct clinically related outcome; five did not have a health intervention or health related outcome; four were reviews of reviews and one was in Spanish.

**Table 1:** Eligibility criteria (February 2011 to March 2012)

**Aspect**	**Criteria**
Scope	PROSPERO will initially include systematic reviews of the effects of interventions and strategies to prevent, diagnose, treat, and monitor health conditions, for which there is a health related outcome.
	The long-term aim is to include details of all ongoing systematic reviews that have a health related outcome in the broadest sense (for example, reviews of risk factors and genetic associations). Reviews of animal studies will not be included.
Review types excluded	Scoping reviews, reviews of reviews, and reviews of methodological issues are not currently included in PROSPERO.
Timing	Registration should take place once the systematic review protocol has been finalised, but ideally before screening studies for inclusion begins. However, during the initial period of operation we will accept registration of reviews that are already underway up to the point of completion of data extraction. Completed reviews should not be registered.
Cochrane Review Protocols	An electronic upload of Cochrane Protocols from the Cochrane library is being developed. To avoid duplication of records, Cochrane protocols should not be registered separately with PROSPERO.
Language	Submissions must be in English.
	If you are in any doubt about the eligibility of your review or the stage of progress please contact crd-register@york.ac.uk for advice.

For the period 1 March 2011 to 21 February 2012 the average administrative turn round time for accepted submissions was 1.0 working day.

#### Published records

**Figure 1:**
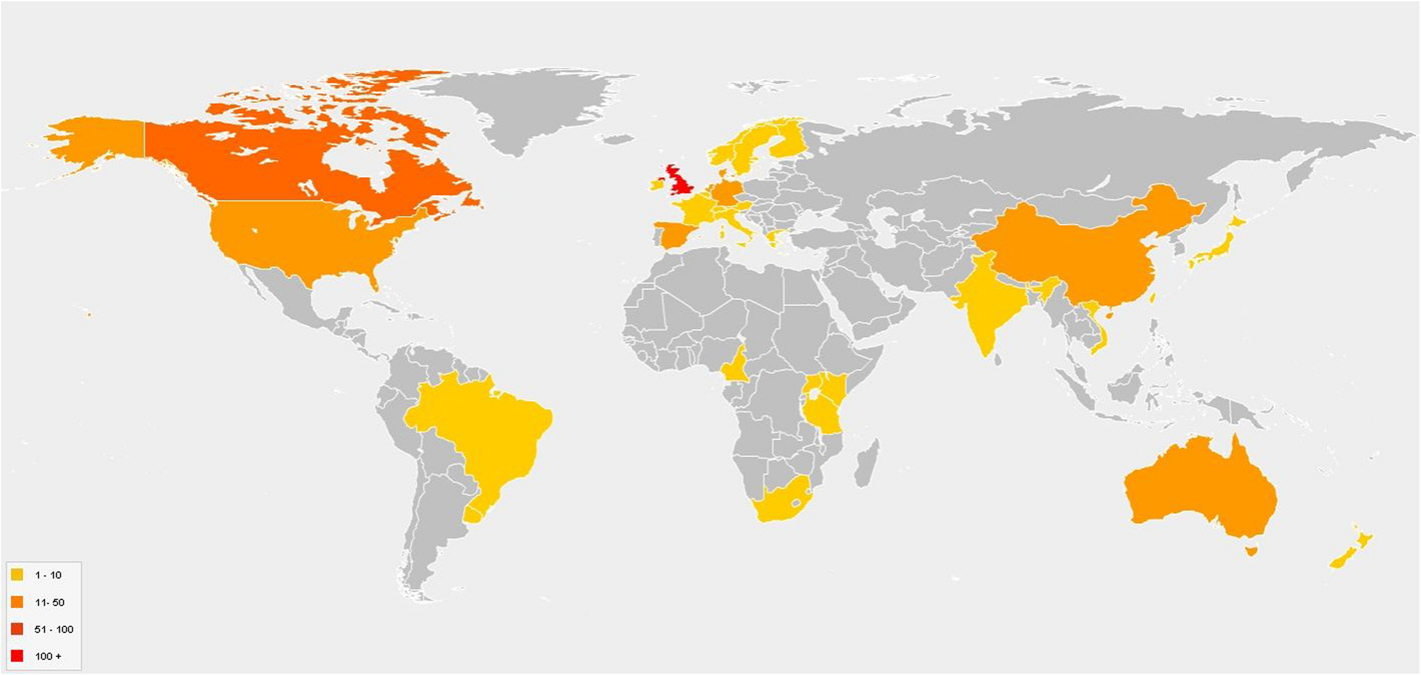
Countries where registered reviews are being undertaken.

On 21 February 2012, 359 systematic reviews were registered on PROSPERO. The reviews were being undertaken in 33 different countries (Figure 1), many of them in collaboration between two or more countries. The 10 countries with the most registrations are listed in Table 2.

**Table 2:** Top ten countries

**Country**	**Sole country undertaking the review**	**Additional reviews being undertaken in collaboration with other countries**	**Total**
England	113	28	141
Canada	38	12	50
United States of America	22	16	38
Australia	22	10	32
Brazil	16	3	19
Netherlands	8	11	19
Scotland	8	9	17
China	12	1	13
Denmark	7	6	13
Germany	9	4	13

All the registrations were in English as other languages are not accepted. All but one of the reviews will be written in English; one will also be available in German, two also in Norwegian and one in Spanish only.

**Figure 2:**
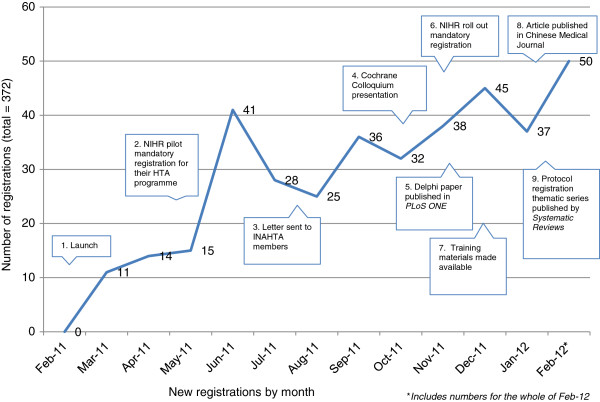
Rate of new registrations.

The overall trend for submission of registrations increased exponentially, but there were a number of peaks in activity that may be explained by a variety of activities (Figure 2):

1. 22 February 2011. Launch events in UK and Canada and press releases sent to all relevant agencies and organizations (for example, INAHTA, G-I-N);

2. 1 May 2011. NIHR piloted mandatory registration for all HTA programme funded reviews, and contacted all those already funded to register if still within acceptance criteria.

3. 27 July 2011. Letters sent to all INAHTA member organisations encouraging support for PROSPERO by making registration part of the funding process.

4. 21 October 2011. Presentation on PROSPERO given at the Cochrane Colloquium in Madrid.

5. 16 November 2011: Paper about the international consultation to establish the minimum dataset published in PLoS ONE [2].

6. Mid-end November 2011. NIHR rolled out mandatory registration across all their other programmes, and contacted all those already funded to register if within acceptance criteria [7].

7. End of November 2011: Website ‘About’ pages revised and expanded; training materials made available to download [8].

8. January 2012: Paper promoting PROSPERO published in prominent Chinese Medical Journal [9].

9. 9 February 2012: New BMC journal Systematic Reviews published a thematic series on ‘The importance of registering systematic reviews’ , including commentaries from Dame Sally Davies (NIHR), Ian Graham (CIHR), the editors of the journal and an article on the nuts and bolts of PROSPERO [10].

The first record to be published on PROSPERO was for a systematic review and multiple treatment meta-analysis of drug-trials for hypertension carried out by the Norwegian Knowledge Centre for the Health Services, which was completed and published in April 2012 [11,12]. The last record published before the first year cut-off was a Joanna Briggs Institute review funded by the Australian Agency for International Development. This review is looking at demand-side financing measures to increase maternal health service utilization and improve health outcomes in low and middle income countries and is due for completion in September 2012 [13].

There were 171 Treatment, 46 Prevention, 40 Service delivery, 36 Diagnostic, 31 Prognostic and 39 ‘Other’ reviews registered on the database (categories selected from a drop-down menu). Funding sources included government agencies (130), institutional (university/hospital) (71), pharmaceutical company (10), miscellaneous other funders (11) and no funding (137). Organisational affiliation included government agencies (4), hospital/medical centers (56), research institutes (82), University/Medical schools (169) and pharmaceutical companies (4). Forty-four gave no organisational affiliation (providing this information is currently optional).

There were 435 registered users who had ‘joined’ but never created a form and 266 who had created but never submitted a form. This included users who had registered more than once, using different email addresses. Those who had never created or submitted a form were asked why in the questionnaire. The reasons given included: interest in seeing the form but not in a position to register a review (for example, team member not lead; Cochrane review; took part in formulating minimum dataset); found their review did not meet the inclusion criteria (for example, it was too far advanced); were considering registering but as yet undecided; were about to submit their form.

### Database usage

**Figure 3:**
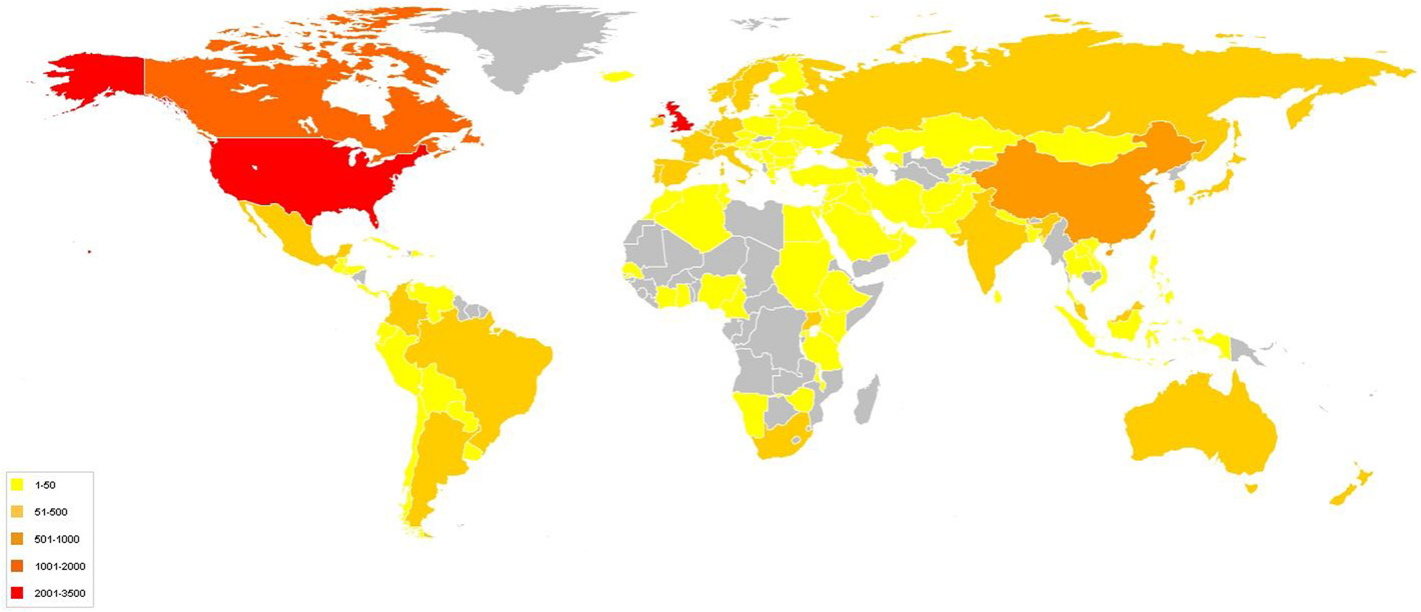
Countries accessing PROSPERO: map.

The total number of hits on the PROSPERO website between 22 February 2011 and 21 February 2012 was 406,730; there were 829,766 page views by 13,607 visitors (Figure 3).

**Figure 4:**
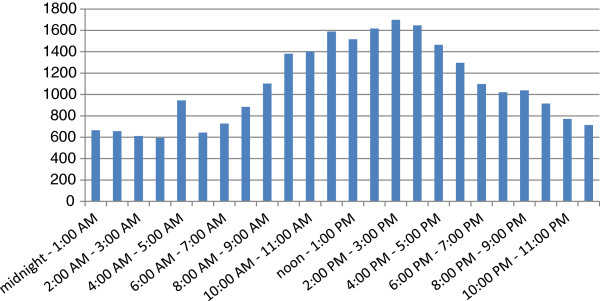
Visitors by time of day (GMT).

Users in 28 identified countries and territories around the world accessed the database (Figure 3). The highest use was from the UK, USA, Canada and China. The international nature of the register was further demonstrated by around the clock access (Figure 4).

The five identifiable websites that referred most visitors were: http://www.crd.york.ac.uk/; http://www.york.ac.uk/; http://www.google.co.uk/; http://www.prisma-statement.org/; http://www.google.com/. The search engines referring most visitors were Google, Bing and Yahoo. The top five search phrases used to find PROSPERO were: prospero; prospero crd; prospero systematic reviews; prospero systematic review; and prospero York.

### User experience

The survey link was sent to the active email accounts of 1,047 registered user accounts of which 48 were duplicate accounts for the same named contact and as the questionnaire blocked more than one response per computer, we anticipated a maximum return of 999. A total of 232 responses were received, giving a response rate of 23%. None of the questions were compulsory so the number of responses per question varied.

Overall, the feedback on functionality and ease of use was positive. Brief details are given here with additional information provided in Additional file 2. The joining process and navigation around the registration form were considered to be easy or very easy by 99% of users. Supporting materials, including guidance, references and links to other resources, were found to be helpful or very helpful by 67% of respondents; most people (177 (82%)) found all or most of the registration fields of relevance to the systematic review protocols they had registered or were likely to register.

The majority of respondents, 176 (81%) had a written protocol for their systematic review before completing the PROSPERO registration form. Of those who did not have a protocol; 42 (19%) used their grant proposal or detailed project description to complete the registration form. In two cases, the protocol was designed using the headings from PROSPERO. Others found that completing the registration form helped improve their protocol by making them formalise less detailed areas. One registrant had split the protocol for a review looking at two different clinical areas, funded by a single grant.

#### Completing the registration form

Most submissions took between 30 minutes and 1 hour to complete; 136 (79%) indicated they completed the registration form in 60 minutes or less. The majority, 167 (87%), considered the time taken to be about right; 24 (12%) felt it took too long and 2 (1%) too short a time. Comments received indicated that for those with a prepared protocol, completion was quick. Where protocols were in a different format completion took longer, but there appeared to be a willingness to change to the PROSPERO format. Some used the registration form as a guide for ‘tidying up’ the protocol and some prepared responses to each of the questions in a separate document, and circulated it to colleagues to ensure it was ready before cutting and pasting into the PROSPERO form. Some felt the time taken depended on the subject of the review, and that completion of the form would become easier with familiarity.

A guideline developer with multiple reviews for each guideline indicated that they were weighing the time/resources involved against the benefits of registration.

Overall, there appeared to be recognition that for registration to be of good quality it needs an adequate amount of time to be spent on it and the protocol, and that the process helped.

Respondents reported that they were ‘impressed with the turnaround time and the very friendly contact’ and the majority rated the turn round time for a decision (121 (97%)) and information provided in correspondence (99 (79%)) as excellent or very good.

All seven respondents who had had a submission rejected said the reason for rejection was made clear in the email response. However, two said eligibility was not clear in the information given in the form or on the PROSPERO website; two did not look at the time; and three said that on reflection the information was available at the time. One commented that the inclusion criteria could be more obvious to site users.

PROSPERO compared favourably with previous experiences of trials registration, being ‘on a par’ with the ANZCTR (Australian New Zealand Clinical Trials Registry) and ‘much easier’ than clinicaltrials.gov. The absence of registration fees was identified as helpful. Although a number of respondents felt that PROSPERO was easier, quicker and more flexible than registering a Cochrane Review protocol, the majority acknowledged that the systems are different, particularly in the editorial process.

Overall, 189 (86%) respondents rated their experience of registering their review protocol details on PROSPERO as excellent or good; 21 (10%) as adequate and 9 (4%) as poor. None of those who rated their experience as poor had actually submitted a form, although eight said they are likely to do so in the future and gave positive responses to other questions in the survey. Thirty-two people had two records published, five had three records published, two had four records published, and one individual had seven reviews registered.

Positive comments were made about the information provided in correspondence, how useful the process was for learning how to write a complete protocol for a systematic review, and satisfaction in knowing your work is out in the public domain. The only negative comments were about email enquiries made which had received no response. This flagged a problem in the system which we believe has subsequently been fixed.

Nearly all the respondents who had created a record or previously submitted a registration form said they were very likely or likely to register a systematic review protocol in the future, 207 (94%). However, three (1%) said they would only register if the commissioner of a review made it a requirement. Comments were received from one person who registered only because it was required, one who was required to register but would have done so any way and one who would do so if the commissioners/funders allow it.

#### General feedback

Survey respondents were invited to make additional further comments or suggestions. Some reiterated their support for the planned broadening of scope for inclusion of systematic reviews beyond those of effects. Others asked for more flexibility within the form while still acknowledging the good intent. Suggestions for improvements to the search facility in the public interface were also given.

The majority of comments supported the principle of protocol registration and PROSPERO. Comments ranged from ‘*A very useful tool for a not-very-experienced reviewer. Thank you*’ . to ‘*the resources/references are fantastic. the idea is fantastic and I will persevere, but the form is initially daunting*’ . Many said ‘*Thank you’* and ‘*Congratulations*’.

## Discussion and conclusions

A main aim of this evaluation exercise was to assess the utility of the registration process, and its ‘fitness for purpose’. Inevitably any survey is limited by the response rate and we cannot make assumptions about the views of non-responders. However, the response rate of 23% is typical for an electronic survey [14,15]. The feedback from users about their experiences has provided reassurance that on the whole the process is working well and has prompted some changes and planned developments to improve the user experience (Additional file 3). Requests to include alternative review types were made in the survey and by separate request; in particular, that reviews of reviews and methodology reviews be accepted. The PROSPERO Advisory Group have since agreed that reviews with a methodological focus, which also include an outcome of direct patient or clinical relevance, should be included in PROSPERO. The advisory group also agreed that systematic reviews of reviews should in future be included in PROSPERO; all other inclusion/exclusion criteria would still apply. One of the most encouraging findings is the range of reviews being registered not only in terms of countries, organisational affiliations and funding but also in countries collaborating on reviews.

Database usage statistics demonstrate the international interest in PROSPERO with high access around the clock and across the week. We are aware that PROSPERO is being routinely searched prior to new reviews being commissioned and, therefore, is already helping to avoid unintended duplication of reviews.

## Competing interests

All the authors are members of the PROSPERO advisory group and have been since the inception of the register. The authors declare they have no other competing interests.

## Authors’ contributions

The decision to undertake an evaluation of utility at one year and the elements to be included was made by members of the PROSPERO Advisory Group. AB drafted the list of statistics to be collected from the website and administrative system and questions to be asked in the user survey. MC, MP and LS made substantial contributions to the lists for data collection and the questionnaire. AB managed the acquisition of the statistical data and survey administration and responses. AB presented the results to the PROSPERO Advisory Group (MC, GD, DG, DM, MP and LS) for interpretation and action. All the authors contributed to the analysis and interpretation of the data. AB and LS produced the first draft of the article and MC, GD, DG, DM and MP critically commented. All the authors have approved the final version submitted.

## Supplementary Material

Additional file 1 Survey questions. Click here for file

Additional file 2 Survey responses. Click here for file

Additional file 3 Modifications made to PROSPERO in response to user survey findings. Click here for file
