# Supplementary material for: PROSPERO at one year: an evaluation of its utility
Source: Syst Rev. 2013 Jan 15;2:4. doi: 10.1186/2046-4053-2-4 (PMC3563608; doi:10.1186/2046-4053-2-4)
Supplement: Additional file 1 — Survey questions. [file 2046-4053-2-4-S1.pdf]

Additional file 1

Survey Questions

1. How would you rate the ease of the 'Join' process, which allowed you to create a user account for PROSPERO?

2. We are interested in why you have completed the 'Join' process for PROSPERO but do not currently appear to have submitted a registration form as Named Contact. (Please tick all that apply)

- *Took part in the Delphi consultation: interested in result*
- *Curious to see registration process*
- *Found my review was not eligible so did not submit*
- *Will be registering in the near future*
- *Another team member took responsibility for registration*

(N.B. Question only sent to emails of registered users who had not submitted a registration form.)

3. How would you rate the ease of navigating around the registration form?

- *Very easy*
- *Easy*
- *Not easy*
- *Difficult*

4. How useful did you find the following supporting materials?

- *Information about field content given in the form*
- *Full information about field content accessed via the ? icon in the form*
- *The 'About PROSPERO' pages on the website*
- *The 'References and resources' provided on the website*
- *The pdf of the Guidance notes for completing the registration form*

5. Please indicate the type of review you are likely to register/have submitted for registration

- *Type of review*
- *Diagnostic*
- *Service Delivery*
- *Prevention*
- *Prognostic*
- *Treatment*
- *Other (please specify)*

6. In the registration form you were asked to indicate the stage of your systematic review as 'started' or 'completed' for each of the options listed below. How relevant do you think these options are for indicating the stage of your systematic review from initial submission to completion of the review?

- *Preliminary searches*
- *Piloting of the study selection process*
- *Formal screening of search results against eligibility criteria*
- *Data extraction*
- *Risk of bias (quality) assessment*
- *Data analysis*

7. On a few submissions, registrants indicated they had started (but not completed) Data extraction, Risk of bias (quality) assessment, and Data analysis.

We anticipated that data analysis would not begin until data extraction had been completed. To inform a review of the timing for acceptance of registrations, we would be interested to know the circumstances in which all these stages are active at the same time. Details of your experience of this and/or your comments are welcome.

8. In general, how relevant were the registration fields to the systematic review protocol you were registering? / are likely to register?

- *All relevant*
- *Mostly relevant*
- *Mostly irrelevant*
- *I cannot remember*

9. If you had any problems deciding what information to enter in which field, please describe the problems.

10. How useful did you find the following technical facilities within the registration form?

- *Highlighting of Required fields*
- *'Save' button on each page*
- *'Validate this page' facility*
- *Ability to print a copy of the form*
- *Ability to upload pdf of search strategy*
- *Ability to upload pdf of protocol*

11. How useful would you find it to be able to do the following?

- *Save a draft form as a pdf file*
- *Save a draft form as a document that could be edited in word processing software*
- *Save the submitted form as a pdf file*
- *Save the submitted form as a document that could be edited in word processing software*

12. How long did it take you to complete the registration form?

- *Up to 30 minutes*
- *30 to 60 minutes*
- *Over 60 minutes*
- *I cannot remember*

13. Did you feel the time taken to complete the registration form was:

- *Response*
- *Too long*
- *About right*
- *Too short*
- *No opinion*

14. Did you have a written protocol for your systematic review before you completed the PROSPERO registration form?

- *Yes*
- *No*

15. Following submission of your registration form, how would you rate the following?

- *The turnaround time for a decision*
- *The information provided in correspondence*

16. If your submission was rejected, was the reason for rejection made clear in the email response?

- *Options*
- *Yes*
- *No*
- *Can't remember*

17. If your submission was rejected, on reflection, was the reason for rejection clear in the information given in the form or on the PROSPERO website?

- *No, not at the time of submission*
- *Yes on checking, the information was available at the time*
- *Did not look at the time*
- *Can't remember*
- *Information on eligibility was not clear at the time*
- *Information currently provided is still not clear*
- *Information is now provided and clear*

18. If you have experience of registering a systematic review protocol or any other piece of research anywhere else, we would be interested to hear your opinion of how PROSPERO compares with other registers.

19. Overall, how do you rate your experience of registering your systematic review protocol on PROSPERO?

- *Excellent*
- *Good*
- *Adequate*
- *Poor*

20. How likely are you to (return and) register a systematic review protocol in the future?

- *Very likely*
- *Likely*
- *Unlikely*
- *Only if the commissioner or funders require it*

21. If you have any further comments or suggestions please make them here or email us at crd-register@york.ac.uk

---
