# Supplementary material for: PROSPERO at one year: an evaluation of its utility
Source: Syst Rev. 2013 Jan 15;2:4. doi: 10.1186/2046-4053-2-4 (PMC3563608; doi:10.1186/2046-4053-2-4)
Supplement: Additional file 2 — Survey responses. [file 2046-4053-2-4-S2.pdf]

Additional file 2

Survey responses

Registering a review

The joining process and navigation around the registration form was considered to be easy or very easy by 99% of registrants.

Of those who had registered as users but not yet submitted a registration form, 39 (45%) said they would register their review in the near future; 26 (30%) found their review was not eligible and so did not submit; 24 (27%) were interested to see the process (three had taken part in the Delphi consultation); and 18 (21%) said another team member took responsibility for registering their review.

Supporting materials such as brief and full guidance about field content, general information about PROSPERO and references and links to other resources are provided on the website and within the registration form. These supporting materials were found to be helpful or very helpful by 67% of respondents; 28% were unaware of them or aware but did not use them. Two respondents suggested that the eligibility criteria could be placed more prominently on the website. One person registering a 'slightly less conventional systematic review' did not find the guidance given really helped. Another person suggested providing a sample of a review protocol.

Type of review

Respondents were asked to indicate the type of review they had or were likely to register. Of the current options: 110 (56%) indicated a treatment review; 33 (17%) prevention; 24 (12%) service delivery; 19 (10%) diagnostic; and 12 (6%) a prognostic review. The numbers were very similar for those who had already submitted a review and those who planned to in the future. A list of 39 'Other' types were given in the free text comments box; of these 20 were felt to fit existing categories. Three were methodology reviews and two reviews of reviews. Five referred to method to be use in conducting the review, e.g. meta analysis or IPD. The remaining nine were epidemiology related.

Stage of progress

Every submission and subsequent revision requires details of the stage of the systematic review by indicating whether key stages of the review have been 'started' or 'completed'. Respondents found these options to be very relevant: Preliminary searches 176 (81%); Piloting of the study selection process 147 (67%); Formal screening of search results against eligibility criteria 192 (88%); Data extraction 187 (86%); Risk of bias (quality) assessment 170 (78%); Data analysis 186 (85%).

A few submissions indicated that data extraction, risk of bias (quality) assessment, and data analysis had been started but not completed. When designing PROSPERO we anticipated that data analysis would not begin until data extraction had been completed. The questionnaire therefore asked for circumstances in which these stages might be active concurrently. Responses included: none; the re-running of searches; delayed receipt of information for inclusion; different parts of a review or reviewers progressing at differing rates; entry of extracted data directly into analysis software; a characteristics table being

compiled during data extraction as part of the analysis; and for qualitative systematic reviews quality assessment, data extraction, and data analysis would be concurrent and iterative.

Relevance of fields

The majority of respondents (177 (82%)) found all or most of the registration fields relevant to the systematic review protocols they had registered or were likely to register. Three commented that they were not well tailored for reviews including qualitative studies and another asked for more flexibility in the information required in fields; specifically primary and secondary objectives.

There were a few reported problems concerning decisions about what information to enter in which field. These related to non-intervention systematic reviews, qualitative systematic reviews and reviews done as part of a multiple strand project, but had not prevented completion and submission of a registration form. Some fields were felt to be a bit redundant (but not named).

Functionality of the registration form

The technical facilities within the registration form were felt to be useful or very useful by the majority of those who were aware of them. One hundred and sixty three (77%) found highlighting of required fields useful or very useful, 44 (20%) did not use or were unaware of the highlighting; one person commented that it could be clearer.

The 'save' button on each page was useful or very useful for 175 (82%); 32 (15%) did not use or were unaware of the save button; a number commented that although the system automatically saves changes when the user exits a field, being able to ‘manually save’ gave good reassurance and the confidence to be able to complete the form in stages.

The 'validate this page' facility, which highlights any required fields that have not yet had any information entered, was useful or very useful to 153 (72%) of respondents.

The ability to print a copy of the form was useful or very useful to 150 (71%) of respondents.

The option of being able to upload pdfs of the search strategy and/or protocol was considered to be very useful or useful by 135 (63%) and 138 (65%) respectively.

The majority of respondents (86%) indicated that being able to save the draft and submitted form as either a pdf file or a document that could be edited in word processing software would be very useful or useful. Reasons given were around facilitating distribution amongst co authors to assist with joint formulation of submissions and subsequent updates/revisions of existing records.
