# Supplementary material for: PROSPERO at one year: an evaluation of its utility
Source: Syst Rev. 2013 Jan 15;2:4. doi: 10.1186/2046-4053-2-4 (PMC3563608; doi:10.1186/2046-4053-2-4)
Supplement: Additional file 3 — Modifications made to PROSPERO in response to user survey findings. [file 2046-4053-2-4-S3.pdf]

Additional file 3

Modifications made to PROSPERO in response to user survey findings

The PROSPERO year one user survey findings reflected a positive experience for those who responded, but also identified some areas for improvement and some for consideration in the next phase of development. The actions taken and future considerations are outlined here.

Scope for inclusion

Inclusion criteria have been made more prominent on the PROSPERO website, and included in the full guidance notes. Future developments to accommodate the inclusion of other types of review will similarly be accompanied by relevant guidance. Examples of information required for each field are given in the full guidance notes and PROSPERO now contains numerous examples which all registrants can access. The PROSPERO 'Help with registration: Inclusion criteria' page on the website has also been revised. [http://www.crd.york.ac.uk/PROSPERO/inclusion_criteria.asp]

Support materials

A few users commented that while supporting information was useful, it was not necessarily presented in the most accessible way. As a result separate tabs for 'About PROSPERO', 'Help with registration', and 'References and resources' have been created and the appropriate information placed under sub-headings into each of these pages. The full guidance is being prepared in alternative formats to suit a range of user preferences.

Type of review

Some respondents selected the 'other' category for type of review because the review fitted in more than one category; as from Nov 2011 it has been possible to make multiple selections. As a result of feedback, additional information about what is expected in each type of review will be added to the guidance. The option of Treatment has been amended to Intervention and the guidance notes revised.

Some supplied an alternative descriptor to those listed, but the majority of these were found to fit within existing options. This reflects a degree of confusion over the meaning of 'type' seen in both submitted registrations and in the user survey. A few misunderstood and detailed the methods to be used, for example IPD; meta-analysis; qualitative. An alternative descriptor to 'type' has been sought but all alternatives had similar potential for misinterpretation.

Responses also identified the potential need to include the addition of a term to cover epidemiological type reviews as an option. However there are a range of terms in use, for example epidemiological could be aetiological or observational; prevalence; risk. The most inclusive and widely understood and used term for this category of reviews is being considered before being added to the list. The categories will need to be reviewed regularly as scope for inclusion broadens.

Stage of review at time of submission

This field is important to users of the database as an indication of the stage the review is at when first registered, and when subsequent amendments and updates are made to the

record. As a result of feedback, an additional option of Started – Yes/No is being added to the field; and the option of Prospective Meta-Analysis removed. Consideration will be given to other changes when a review of the dataset is undertaken.

Relevance of fields

The dataset currently required was agreed for the initial inclusion of reviews of the effects of interventions, with the understanding that modifications or alternative templates would probably be required as the scope for inclusion expanded. The majority of respondents found all or most of the registration fields of relevance to the systematic review protocols they had registered or were likely to register. A few respondents felt the fields were not well tailored for some reviews, for example when including qualitative studies. Given the range and variety of reviews now registered, the system would appear to have a good degree of flexibility, supported by a pragmatic approach to inclusion by the administration team.

The terminology and separation of 'primary' and 'secondary' objectives, was questioned for being related to trials rather than systematic reviews. However, as this is the language used by The Cochrane Collaboration, in the PRISMA statement and agreed through international consultation, it was not felt necessary to change at this time. The advisory group also felt the separation of the terms is appropriate for the current inclusion criteria, as it requires the focus of the review to be clearly stated a priori, with the option of including secondary objectives if required.

A full evaluation of the required dataset will be undertaken in the future and relevance of fields considered during the stepped approach to expansion of the scope for inclusion.

Functionality of the registration form

Enabling a copy of the draft and submitted form to be saved as a pdf file and a word processing document to facilitate editing by multiple authors is a feature that will be added to the registrant interface as soon as this can be technically achieved. (In the meantime, clicking on the Print review button, then viewing the list of printer options should identify a way of saving the form as a pdf.)

General issues

Five respondents commented that they had not received a response to an email enquiry. None supplied contact details so we are unable to follow them up individually. However, all enquiries received at CRD-register@york.ac.uk (and at alison.booth@york.ac.uk) have been responded to. Response time is generally same or next day. A note has been added to the PROSPERO contact page saying: "We aim to respond to all enquiries within 5 working days. We have recently experienced some problems receiving emails, so if after 5 days you have not heard from us, please try emailing crd@york.ac.uk, call, fax or write to us."

Finally, a number or respondents commented that they could not remember specific aspects of the registration process well enough to comment on the experience. However many also said they would be willing to give feedback at the time of registration if this option was made available. All registrants are now invited to take part in a short on-line survey via a link in the email confirming acceptance or rejection of a submission.
